# Regionalisation of the mouse visceral endoderm as the blastocyst transforms into the egg cylinder

**DOI:** 10.1186/1471-213X-7-96

**Published:** 2007-08-16

**Authors:** Aitana Perea-Gomez, Sigolène M Meilhac, Karolina Piotrowska-Nitsche, Dionne Gray, Jérôme Collignon, Magdalena Zernicka-Goetz

**Affiliations:** 1 Wellcome Trust/Cancer Research UK Gurdon Institute, University of Cambridge, Tennis Court Road, Cambridge, CB2 1QR, UK; 2 Institut Jacques Monod CNRS UMR 7592, Université Paris 6 and Paris 7, 2 place Jussieu, 75005 Paris, France; 3 Department of Developmental Biology, CNRS URA2578, Pasteur Institute, 25 rue du Dr Roux, 75015 Paris, France; 4 Division of Neuroscience Yerkes National Primate Research Center, Emory University School of Medicine, 954 Gatewood Rd., Atlanta, Georgia 30329, USA; 5 Department of Anatomy, University of Cambridge, Downing Street, Cambridge CB2 3DY, UK

## Abstract

**Background:**

Reciprocal interactions between two extra-embryonic tissues, the extra-embryonic ectoderm and the visceral endoderm, and the pluripotent epiblast, are required for the establishment of anterior-posterior polarity in the mouse. After implantation, two visceral endoderm cell types can be distinguished, in the embryonic and extra-embryonic regions of the egg cylinder. In the embryonic region, the specification of the anterior visceral endoderm (AVE) is central to the process of anterior-posterior patterning. Despite recent advances in our understanding of the molecular interactions underlying the differentiation of the visceral endoderm, little is known about how cells colonise the three regions of the tissue.

**Results:**

As a first step, we performed morphological observations to understand how the extra-embryonic region of the egg cylinder forms from the blastocyst. Our analysis suggests a new model for the formation of this region involving cell rearrangements such as folding of the extra-embryonic ectoderm at the early egg cylinder stage. To trace visceral endoderm cells, we microinjected mRNAs encoding fluorescent proteins into single surface cells of the inner cell mass of the blastocyst and analysed the distribution of labelled cells at E5.0, E5.5 and E6.5. We found that at E5.0 the embryonic and extra-embryonic regions of the visceral endoderm do not correspond to distinct cellular compartments. Clusters of labelled cells may span the junction between the two regions even after the appearance of histological and molecular differences at E5.5. We show that in the embryonic region cell dispersion increases after the migration of the AVE. At this time, visceral endoderm cell clusters tend to become oriented parallel to the junction between the embryonic and extra-embryonic regions. Finally we investigated the origin of the AVE and demonstrated that this anterior signalling centre arises from more than a single precursor between E3.5 and E5.5.

**Conclusion:**

We propose a new model for the formation of the extra-embryonic region of the egg cylinder involving a folding of the extra-embryonic ectoderm. Our analyses of the pattern of labelled visceral endoderm cells indicate that distinct cell behaviour in the embryonic and extra-embryonic regions is most apparent upon AVE migration. We also demonstrate the polyclonal origin of the AVE. Taken together, these studies lead to further insights into the formation of the extra-embryonic tissues as they first develop after implantation.

## Background

Survival of the mammalian embryo *in utero* requires the early establishment of an extra-embryonic interface to provide nutrients and signals for the maintenance of pluripotent embryonic precursors. Extra-embryonic tissues are the first to differentiate during mouse development (reviewed in [1]). Recent work has demonstrated that these tissues also play an essential role in anterior-posterior (AP) patterning during early post-implantation development (reviewed in [2]). Understanding the formation and growth of extra-embryonic tissues is therefore essential for further studies of the cellular and molecular mechanisms underlying these functions.

Segregation of embryonic and extra-embryonic lineages is completed at the time of implantation. In the E4.5 blastocyst, three tissues can be distinguished. The trophectoderm, which allows attachment of the conceptus to the uterine wall, is composed of a mural part surrounding the blastocoelic cavity, and a polar part in contact with the epiblast. Pluripotent epiblast cells will form all foetal tissues. Finally the primitive endoderm, lining the blatocoelic surface of the epiblast, will give rise to the visceral endoderm (VE), and to the parietal endoderm, which migrates away along the mural trophectoderm. Primitive endoderm and epiblast both derive from the inner cell mass (ICM) of the E3.5 blastocyst.

After implantation major morphogenetic changes have occurred. The conceptus now resembles an elongated cup-shaped structure called the egg cylinder. This comprises two regions. At the distal pole, the embryonic region is formed by the epiblast and the overlying VE. The extra-embryonic region, at the proximal pole, contains the extra-embryonic ectoderm, a derivative of the polar trophectoderm, covered by VE. Because of the relative inaccessibility of the mouse conceptus, little is known about the dynamics of these morphological changes. In the E4.5 blastocyst, the epiblast is already present and covered by the primitive endoderm thus defining the embryonic region. In contrast, the formation of the extra-embryonic region is puzzling since the polar trophectoderm in the E4.5 blastocyst is a single layer of cells not in contact with the primitive endoderm. Differential rates of proliferation between polar and mural trophectodermal cells and uterine mechanical constraints have been proposed to explain the formation of the extra-embryonic ectoderm and its engulfment by the VE [3]. However, the detailed cellular architecture of the polar trophectoderm is poorly described, and other scenarios for the formation of the extra-embryonic ectoderm involving changes in cell shape or cell arrangement cannot be discounted.

Both the embryonic and extra-embryonic regions are covered by the VE. These two VE regions can be distinguished by their cellular architecture [4,5] and by differential gene expression [6-8] at E5.5. Cell labelling experiments have shown that the VE precursors at both ends of the animal-vegetal axis of the blastocyst contribute to both embryonic and extra-embryonic regions of the egg cylinder, although cells from one of these two ends contribute more cells to the embryonic region [9]. These studies also revealed that the pattern of VE cell growth between these regions is different: while extra-embryonic VE clones were more coherent, the embryonic clones were more dispersed and consisted of small groups of scattered cells at E6.5 [9,10]. However, it has remained unknown whether the embryonic and extra-embryonic regions constitute distinct cellular compartments which do not mix, at the time of their formation shortly after implantation. Moreover it remains to be understood how early after implantation VE cells begin to behave differently in embryonic and extra-embryonic regions and how the pattern of their growth relates to the appearance of histological and molecular differences in these regions and to the formation and migration of the AVE.

Extra-embryonic tissues are involved in tightly regulated reciprocal interactions with the epiblast leading to the establishment of AP polarity at E5.5 (reviewed in [2]). Nodal, a member of the TGFβ superfamily, is required in the epiblast for the specification of a population of distal VE cells [11]. At E5.5 these cells are characterised by a columnar shape, which provides a visible landmark known as the visceral endoderm thickening (VET) [5], and by the expression of a specific panel of genes encoding transcription factors like the homeoprotein Hhex or secreted proteins like the TGFβ antagonists Cerberus-like 1 (Cer1) and Lefty1 [12-14]. The anterior pole of the embryo is defined by the asymmetric migration of these cells towards one side of the egg cylinder, where they form the AVE [15,16]. Some genes marking the AVE at E5.5 like *Hhex *and *Lefty1 *are already expressed in the E3.5 ICM and in the E4.5 primitive endoderm though with different patterns [7,15,17,18]. These findings raise the question of the origin of the AVE. AVE cells could arise from a single precursor at the blastocyst stage indicating an early segregation of this lineage during pre-implantation stages. Alternatively the AVE could be formed by non-clonally related VE cells induced between E3.5 and E5.5.

The aim of our study has been to analyse the formation of the embryonic and extra-embryonic regions as the blastocyst transforms into the egg cylinder. As a first step we have characterised the morphology of E4.7-E5.0 conceptuses and observed an actin-rich depression in the extra-embryonic ectoderm at the time of the formation of the extra-embryonic region. We have used the technique of single cell labelling in the blastocyst to target VE precursors in the ICM surface [9] and analyse their progeny at E5.0, E5.5 and E6.5. We show that as early as E5.0 the embryonic and extra-embryonic regions of the VE do not correspond to distinct cellular compartments, and that growth is not restricted across their junction even after the appearance of histological and molecular differences at E5.5. However we observe distinct patterns in embryonic and extra-embryonic VE regions from the time of AVE migration, when AP polarity is visible. Finally, using two criteria to identify the AVE, the VET and the activity of a *Cer1-*GFP transgene, we demonstrate for the first time the polyclonal origin of this anterior signalling centre.

## Results

### Morphological steps of formation of the extra-embryonic region at the time of implantation

After implantation, two regions are distinguishable within the conceptus. Distally, the embryonic region is formed by the epiblast and the overlying VE, whereas the extra-embryonic region proximally contains the extra-embryonic ectoderm covered by VE. In contrast before implantation these two regions cannot be identified. How are they formed?

**Figure 1:**
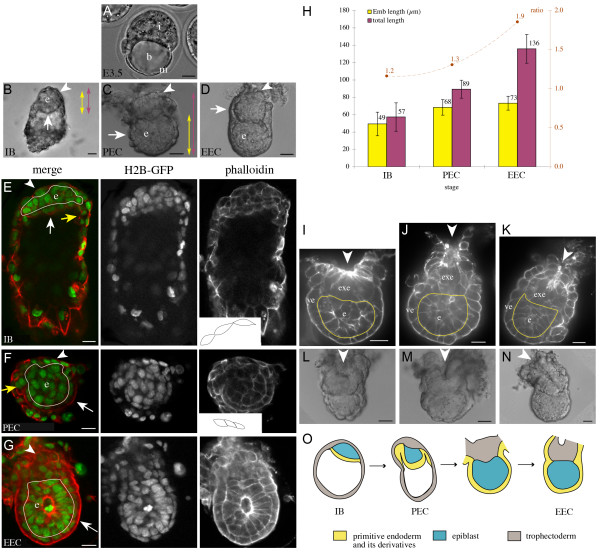
**Formation of the extra-embryonic region of the egg cylinder**. (A-D) Bright field images of mouse conceptuses, before implantation at E3.5 (A), and after implantation at E4.7-E5.0 (B-D). Arrowheads point to the polar trophectoderm, later called extra-embryonic ectoderm (from pre-egg cylinder stage). White arrows show the visceral endoderm. b, blastocoel ; e, epiblast ; EEC, early egg cylinder; i, inner cell mass (ICM) ; IB, implanted blastocyst ; m, mural trophectoderm; PEC, pre-egg cylinder. (E-G) Phalloidin-Texas red staining (red) of *H2B-GFP *(green) transgenic conceptuses, showing respectively the membranes and nuclei of cells. Confocal sections of conceptuses at the implanted blastocyst (IB in E), pre-egg cylinder (PEC in F) and early egg cylinder (EEC in G) stages are shown. Yellow arrows show the parietal endoderm. The contour of the epiblast is highlighted in white. Insets show the contour of 3 neighbouring cells of the polar trophectoderm. (H) Measurements of the embryonic region (Emb) and total length of conceptuses at E4.7-E5.0, as defined by the coloured double arrows in B-C. The average number is shown and error bars indicate the standard deviation from 12 implanted blastocysts, 18 pre-egg cylinder and 24 early egg cylinder conceptuses. The ratio between the two lengths is represented in brown. pc, proamniotic cavity. Conceptuses are oriented with the proximal and distal poles respectively up and down. (I-K) Confocal sections of E4.8-E5.0 conceptuses stained with phalloidin-Texas red, showing the distribution of actin. Arrowheads point to an enrichment of actin in a folded region of extra-embryonic ectoderm (exe). The contour of the epiblast is highlighted in yellow. ve, visceral endoderm. (L-N) Bright field images of E4.8-E5.0 conceptuses. Arrowheads point to the folding of the polar trophectoderm. (O) Schematic summary showing the characteristics of conceptuses ordered by stage during the formation of the extra-embryonic region. The polar trophectoderm (grey) thickens and folds, whereas the primitive endoderm (yellow), which initially covers the epiblast (implanted blastocyst stage) and mural trophectoderm (pre-egg cylinder stage), engulfs the extra-embryonic ectoderm at the early egg cylinder stage. Scale bars 20 μm.

To answer this question we have recovered conceptuses directly from the crypts of the uterus at different hours on the 5^th ^day of gestation, at the time of implantation. We have analysed their morphological changes based on the staining with phalloidin-Texas red in the *H2B-*GFP transgenic line, to visualise cell borders and nuclei. Three stages of conceptuses were identified, depending on their morphology and on the time of recovery. At the implanted blastocyst stage, primitive endoderm cells, in contact with the blastocoel, were clearly distinct from epiblast cells (white arrow Fig. 1B, E). At this stage some primitive endoderm cells, known as parietal endoderm, were observed on the inner surface of the mural trophectoderm (yellow arrow, Fig. 1E). These embryos were recovered between 3–7pm, and therefore correspond to E4.7. At the pre-egg cylinder stage, a thickening of the polar trophectoderm could be observed. By this stage this cell layer had been rearranged from a squamous (arrowhead and inset Fig. 1E) to a cuboidal epithelium (arrowhead and inset Fig. 1F). When several layers can be identified in this tissue, it takes the name of extra-embryonic ectoderm (arrowhead Fig. 1C). The VE was clearly visualised at this stage and was curved around the epiblast (white arrow Fig. 1F). Pre-egg cylinder stage conceptuses were recovered between 6–9 pm, and therefore correspond to E4.8. It was only at the early egg cylinder stage, that we observed the VE covering the extra-embryonic ectoderm (Fig. 1D). At the early egg cylinder stage, some conceptuses showed evidence of a forming proamniotic cavity, as judged by the occasional presence of a central apoptotic epiblast cell (Fig. 1G). Early egg cylinder conceptuses were recovered between 9 pm–3 am, and therefore correspond to E5.0. These observations show that the extra-embryonic region forms by "thickening" of the polar trophectoderm at the pre-egg cylinder stage, and that it is covered by the VE only from the early egg cylinder stage onwards. In contrast the primitive endoderm already covers the epiblast from the implanted blastocyst stage, thus defining the embryonic region.

To confirm in living embryos the sequence of events described on fixed material, we have performed in vitro cultures. These experiments indicate that implanted blastocysts may develop into pre-egg cylinder conceptuses, as shown by the change in morphology of the polar trophectoderm from a squamous to a cuboidal epithelium (Additional file 1A-B). In addition, pre-egg cylinder conceptuses may develop into early egg cylinder conceptuses as shown by the appearance of several layers of polar trophectoderm cells that become covered by the VE (Additional file 1C-F). Therefore the sequence of stages from implanted blastocyst to pre-egg cylinder and from pre-egg cylinder to early egg cylinder applies to living embryos.

In addition to morphological changes, we have analysed the size of E4.7-E5.0 conceptuses (Fig. 1H). We found that, following the formation of the extra-embryonic region, the total (proximal-distal) length increased about 2.5 fold reaching 136 μm (Standard deviation, SD = 17 μm), whereas the embryonic region, from the distal tip of the conceptus to the proximal end of the epiblast, grew less, from 49 μm (SD = 14 μm) to 73 μm (SD = 8 μm). Consistently, the ratio between the total and embryonic lengths increased from 1.2 at the implanted blastocyst stage to 1.3 at the pre-egg cylinder stage and 1.9 at the early egg cylinder stage. These results indicate that the extra-embryonic region becomes a significant part of the conceptus after pre-egg cylinder stage.

To get insight into the mechanism of formation of the extra-embryonic region, we have analysed in more detail the extra-embryonic ectoderm at pre-egg cylinder and early egg cylinder stages. We found that 45% of pre-egg cylinder (n = 7) and 70% of early egg cylinder conceptuses (n = 21) showed intense phalloidin staining at the proximal pole of the extra-embryonic ectoderm, indicative of a localised enrichment of actin, associated with a slight (Fig. 1I) or more pronounced (Fig. 1J–K) depression of the tissue. This depression was also visible in transmitted light (Fig. 1L–N). In the remaining early egg cylinder conceptuses (n = 8, 30%), we did not detect any actin-enrichment or depression of the extra-embryonic ectoderm (Fig. 1G). However, we noticed that these conceptuses were significantly bigger in size, with a total length of 148 μm (SD = 15 μm), compared to 122 μm (SD = 20 μm) and some of them (50%) showed evidence of a forming proamniotic cavity. This indicates that the actin-rich depression of the extra-embryonic ectoderm is transient and that this feature is lost as the conceptus develops further. Taken together, these observations suggest a new descriptive model for the formation of the extra-embryonic region. After the initial thickening, there is a folding of the extra-embryonic ectoderm which may involve actin. The overlying VE is carried along during this process. This results in the extra-embryonic ectoderm being engulfed by the VE, thereby marking the transition from blastocyst to egg cylinder (Fig. 1O).

### The junction between embryonic and extra-embryonic VE regions is not a compartmental barrier during egg cylinder growth

The VE covers both the embryonic and extra-embryonic region. However VE cells display distinct characteristics in these regions, in terms of cell shape [4] and gene expression [6]. Such differences have been shown from the time of induction of the AVE [5,7], whereas at the early egg cylinder stage we have observed that differences in the shape of VE cells between both regions are less pronounced (Fig. 1G, K). Do the embryonic and extra-embryonic regions correspond to distinct cellular compartments at the time of their formation? Is growth restricted across the embryonic/extra-embryonic junction during egg cylinder development?

We have used a cell labelling approach to address these issues. Precursor cells were labelled before implantation, in the E3.5 blastocyst. We have microinjected mRNA encoding fluorescent proteins into single cells at the surface of the ICM, in order to target preferentially VE precursors [9]. Labelled blastocysts were transferred into the uterus and recovered after implantation at E4.7-E5.0, E5.5 and E6.5 (Additional file 2A). Only E4.7-E5.0 conceptuses which had a clearly visible extra-embryonic region (early egg cylinder stage) were considered for further analysis. On average, 8 (SD = 5) VE labelled cells per conceptus were observed at the early egg cylinder stage (n = 24), 39 (SD = 20) at E5.5 (n = 48) and 154 (SD = 64) at E6.5 (n = 31).

**Figure 2:**
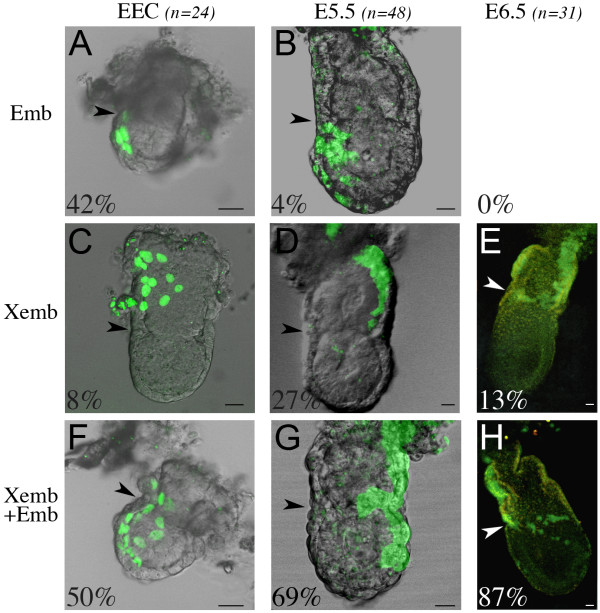
**Lack of growth restriction between the embryonic and extra-embryonic regions of the VE**. Examples of labelled conceptuses recovered after microinjection of single cells at the surface of the ICM at the blastocyst stage. They are shown as a fluorescent projection of a confocal z series merged with a transmitted light section, at the early egg cylinder (EEC) stage (A, C, F), E5.5 (B, D, G) and E6.5 (E, H). For each stage, the number of labelled conceptuses considered (n) and the percentage of those with colonisation of the embryonic region only (Emb, A-B), the extra-embryonic region only (Xemb, C-E) or both (Xemb+Emb, F-H) is indicated. Arrowheads point to the embryonic/extra-embryonic junction. Conceptuses are oriented with the anterior pole on the left, whenever it can be identified. Scale bars 20 μm.

The contribution of VE labelled cells to the embryonic and extra-embryonic regions was analysed. These two VE regions have a similar size, in terms of cell numbers, both at the early egg cylinder stage, when the extra-embryonic region has formed, and at E5.5 (data not shown). Labelled conceptuses at the early egg cylinder stage, E5.5 and E6.5 were classified as embryonic (Fig. 2A–B), extra-embryonic (Fig. 2C–E) or extra-embryonic and embryonic when both regions were colonised (Fig. 2F–H). At all stages examined a majority of conceptuses had labelled cells contributing to both regions (50% at the early egg cylinder stage, 69% at E5.5 and 87% at E6.5). These percentages are significantly higher than the rate of blastocysts with more than one labelled cell after microinjection (see Methods), indicating that the double contribution to embryonic and extra-embryonic regions is a clonal event. This indicates that many VE precursors at E3.5 are not restricted in their ability to contribute to the embryonic or extra-embryonic regions.

The proportion of conceptuses with contribution of labelled cells to both regions significantly increased between the early egg cylinder stage and E6.5 whereas the proportion of labelled conceptuses with exclusive contribution to the embryonic region significantly decreased during the same period (Chi-square test, p < 0.05). This suggests that as the embryo develops, labelled cells that were located exclusively in the embryonic region at the early egg cylinder stage grow and span the embryonic/extra-embryonic junction to colonise the adjacent region. These results indicate that as soon as they can be identified at the early egg cylinder stage the embryonic and extra-embryonic domains of the VE do not correspond to distinct cellular compartments. Moreover there is no restriction of growth between these two regions even after E5.5, when histological and molecular differences have been shown between embryonic and extra-embryonic VE regions [5,7].

### Shift in orientation of VE clusters at the embryonic/extra-embryonic junction after AVE migration

**Figure 3:**
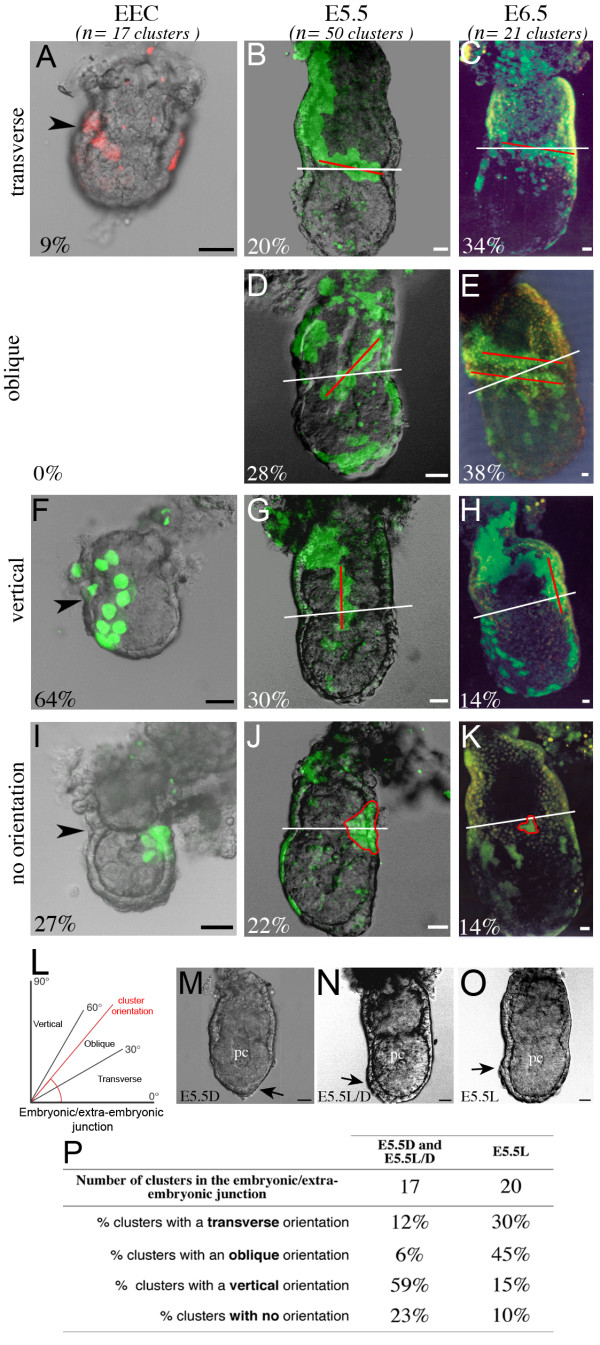
**Shift in orientation of VE clusters at the embryonic/extra-embryonic junction**. (A-K) Examples, shown as a fluorescent projection of a confocal z series merged with a transmitted light section, of VE clusters with a transverse orientation (A-C), oblique orientation (D-E), vertical orientation (F-H) and with no specific orientation (I-K) at the level of the embryonic/extra-embryonic junction at the early egg cylinder (EEC) stage (A, F, I), E5.5 (B, D, G, J) and E6.5 (C, E, H, K). The orientation of clusters at E5.5 and E6.5 is indicated by a red line and that of the embryonic/extra-embryonic junction by a white line. Arrowheads indicate the embryonic/extra-embryonic junction at the early egg cylinder stage. The contour of clusters with no specific orientation is outlined by a red line. For each stage, the number of clusters considered (n) and the percentage of clusters with a given orientation are shown. The number of conceptuses with clusters of labelled cells at the embryonic/extra-embryonic junction considered is 17 at the early egg cylinder stage, 36 at E5.5 and 21 at E6.5. Some conceptuses have several clusters at the embryonic/extra-embryonic junction. (L) Schematic representation of the classification of junction clusters according to the value of the angle between the orientation of the cluster (red line) and that of the embryonic/extra-embryonic junction. (M-O) Bright field images of conceptuses at E5.5. Black arrows show the thickening of the visceral endoderm (VET), which indicates the position of the anterior pole, distally (D), laterodistally (L/D) or laterally (L). (P) Summary table of the orientation of junction clusters at E5.5 according to the position of the VET. Scale bars 20 μm.

Given the lack of growth restriction across the embryonic/extra-embryonic junction, we have investigated whether VE cells at the junction between these regions show specific behaviour. We have focussed on clusters of labelled cells which abut or cross the embryonic/extra-embryonic junction ("junction clusters"). Note that some conceptuses have more than one such cluster. We have noticed that junction clusters display specific shapes. They were classified according to their orientation. Transverse clusters were parallel to the junction and extended either in the embryonic or extra-embryonic region (Fig. 3A–C); some clusters were oblique (Fig. 3D–E); vertical clusters were parallel to the proximal-distal axis of the conceptus (Fig. 3F–H). At E5.5 and E6.5 the angle between the orientation of the cluster and that of the embryonic/extra-embryonic junction was measured, to define the categories (Fig. 3L). At the early egg cylinder stage, clusters were categorised qualitatively as they contained fewer cells and as the embryonic/extra-embryonic junction may not be straight. At all stages when clusters had a rounded shape they were considered as not oriented (Fig. 3I–K).

At the early egg cylinder stage, most "junction clusters" were vertical (64%, Fig. 3F), and only a minority showed a transverse orientation (9%, Fig. 3A). This suggests that between E3.5 and the early egg cylinder stage, VE cells tend to grow along the proximal-distal axis as the egg cylinder elongates, with disregard to the embryonic/extra-embryonic junction. In contrast transverse and oblique orientations were more frequent at E5.5 and E6.5 (48 and 72% respectively, Fig. 3B, C, D, E). These observations indicate that the orientation of clusters at the junction between the embryonic and extra-embryonic region is shifted, such that it becomes preferentially parallel to the junction rather than to the proximo-distal axis of the egg cylinder.

To clarify the timing at which this shift takes place, we have staged E5.5 egg cylinders according to the position of the migrating AVE. We have used the published nomenclature, based on the distal (E5.5D, Fig. 3M), laterodistal (E5.5L/D, Fig. 3N) and lateral (E5.5L, Fig. 3O) position of the VET [5]. More than half of the "junction clusters" were vertical (59%) at E5.5D and L/D (Fig. 3P), similarly to the situation at the early egg cylinder stage. In contrast at E5.5L most "junction clusters" showed transverse or oblique orientations (75%). These results suggest that the orientation of junction clusters is shifted after AVE migration.

We have investigated whether the orientation of junction clusters is different at the anterior and posterior poles after AP polarity becomes visible at E5.5L and E6.5. The majority of vertical clusters appeared to be located in the posterior third of the egg cylinder (71%, n = 7), whereas most transverse clusters were in anterior or lateral positions (94%, n = 16). This suggests that the shift in orientation of junction clusters is more pronounced at the anterior pole. This may be a consequence of the movement of AVE cells.

### VE cells in the embryonic and extra-embryonic regions show distinct growth modes from E5.5

Though the embryonic and extra-embryonic regions do not correspond to distinct cellular compartments, the appearance of distinct histological and molecular properties from E5.5 correlates with specific cell patterns at the junction. Previous work had indicated that different growth modes distinguish each VE region so that cells in the extra-embryonic region grow as more coherent patches while those in the embryonic region grow in a more dispersive way by E6.5 [9]. We have investigated in detail the time of appearance of these distinct growth modes during egg cylinder growth.

**Figure 4:**
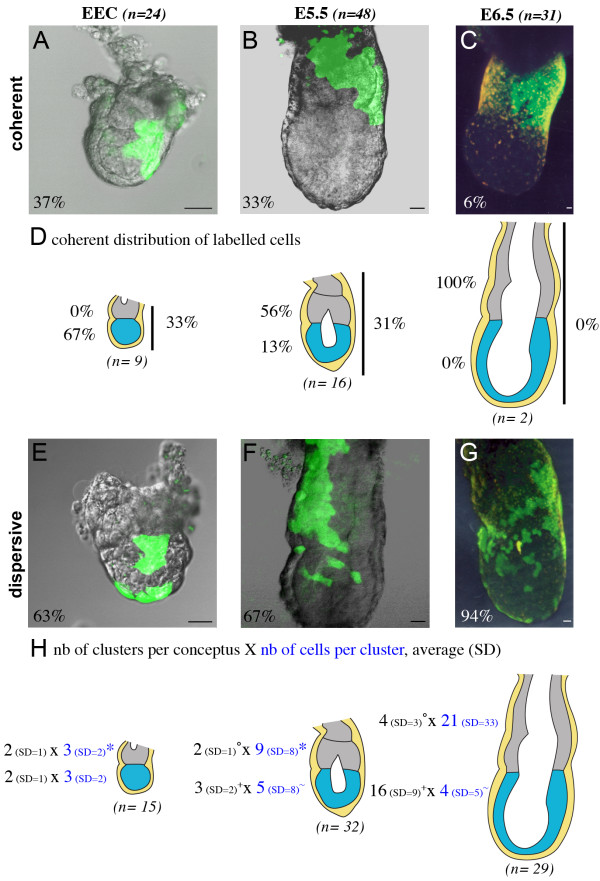
**Cell dispersion in the VE of the embryonic region specifically increases after E5.5**. Examples, shown as a fluorescent projection of a confocal z series merged with a transmitted light section, of coherent (A-C) and dispersive (E-G) distributions of labelled cells at the early egg cylinder (EEC) stage (A, E), E5.5 (B, F) and E6.5 (C, G). For each stage, the number of conceptuses considered and the percentage of coherent and dispersive cases are indicated. (D) Schematic representation of the localisation of labelled cells in coherent distributions. The number of conceptuses considered at each stage is indicated (n) and the percentage of cases with labelled cells in the extra-embryonic region (grey), the embryonic region (blue) or spanning both (vertical bar) is shown. (H) Schematic representation of the characteristics of dispersive distributions of labelled cells in the extra-embryonic region (grey) or embryonic regions (blue). The number of conceptuses considered at each stage (n) is indicated. The average and the standard deviation of the number of clusters per conceptus are shown in black. The number of conceptuses considered with clusters in the extra-embryonic and embryonic regions respectively is 9 and 12 at the early egg cylinder stage, 20 and 21 at E5.5, 26 and 27 at E6.5. Some conceptuses have both extra-embryonic and embryonic clusters. The average and the standard deviation of the number of cells per clusters are shown in dark blue. The number of clusters considered in the extra-embryonic and embryonic regions respectively is 14 and 21 at the early egg cylinder stage, 40 and 53 at E5.5, 92 and 431 at E6.5. *,°,+,~ are significantly different (Mann-Whitney test, p < 0.05). nb, number; SD standard deviation. Scale bars 20 μm.

We have compared the dispersion of labelled VE cells in the embryonic and extra-embryonic regions at the early egg cylinder stage, E5.5 and E6.5. The labelled conceptuses were classified as "coherent" and "dispersive" depending on whether labelled cells form a unique continuous population ("coherent", Fig. 4A–C), or whether distinct clusters of one or more labelled VE cells were separated from the rest of the clone by at least one non-labelled VE cell ("dispersive", Fig. 4E–G).

We have found that while at the early egg cylinder stage and E5.5 around two thirds of the labelled conceptuses were "dispersive" (Fig. 4E, F) and one third were "coherent" (Fig. 4A, B), at E6.5 the vast majority of cases were "dispersive" (94%, Fig. 4G). This indicates that cell mixing in the VE is increased between E5.5 and E6.5. Coherent distributions of labelled cells can be found in the embryonic region of early egg cylinder and E5.5 conceptuses, whereas they are restricted to the extra-embryonic region at E6.5 (Fig. 4D). This suggests that coherent growth of VE cells becomes specific to the extra-embryonic region, between the early egg cylinder stage and E6.5.

To further characterise the dispersion of VE cells we have compared the number, the size and the position of the clusters per labelled conceptus. At the early egg cylinder stage and E5.5, the number of clusters in the embryonic and extra-embryonic regions was similar and relatively low (2 or 3 clusters, Fig. 4H). In contrast between E5.5 and E6.5 cell dispersion in the VE was significantly enhanced, in particular in the embryonic region where the number of clusters significantly increased (Mann-Whitney test, p < 0.005). After E5.5, dispersion also occurred in the extra-embryonic region, yet to a lesser extent (Mann-Whitney test, p < 0.05). Taken together these results suggest that it is only after E5.5, when the AVE has initiated its migration, that different growth modes can be distinguished in the embryonic and extra-embryonic regions, such that dispersion of VE cells is more pronounced in the embryonic region.

### Anterior visceral endoderm cells have a polyclonal origin

We have shown that the embryonic region of the VE is characterised by extensive cell mixing. Yet a group of cells in this region, the AVE, shares specific properties such as a columnar cell shape and expression of specific markers including *Lefty1 *and *Cer1*. In addition, it has been shown that *Lefty1 *is already expressed in some ICM cells of the blastocyst [17], raising the question of whether AVE cells have a common precursor in the blastocyst. Alternatively the AVE may arise from distinct mixed clones.

**Figure 5:**
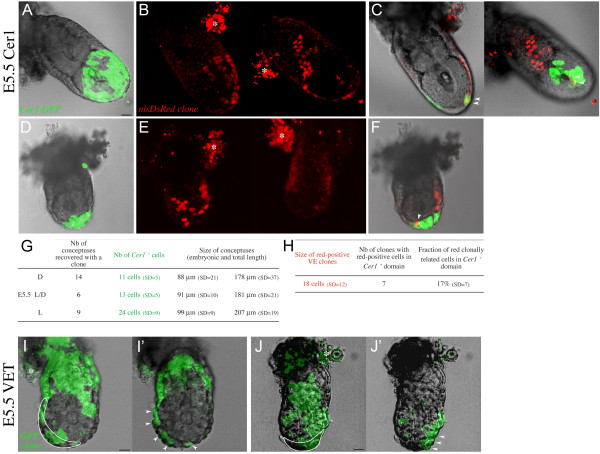
**Polyclonal origin of the Anterior Visceral Endoderm (AVE) region. **(A-H) The *Cer1-GFP *transgenic line is used as a marker of the AVE region. Examples of two conceptuses (A-C and D-F) at E5.5 containing a red-positive clone in the VE. (A, D) *Cer1-GFP *pattern indicates the embryonic stage, respectively E5.5L and E5.5D. Green fluorescent projection of a confocal z series merged with a transmitted light section are shown. (B, E) VE clones labelled with a red nuclear marker. The two sides of the conceptus are shown as a red fluorescent projection of a confocal z series. Asterisks indicate autofluorescence of the parietal endoderm. (C, F) Merged confocal sections of the conceptuses, showing the colocalisation (arrowheads) of the transgenic green and clonal red markers. (G) Summary of the characteristics of conceptuses with a given *Cer1-GFP *pattern. Average numbers are given. D, distal ; L/D, laterodistal; L, lateral; nb, number. (H) Summary of the characteristics of red-positive clones in the transgenic line. Average numbers are given. VE, visceral endoderm. (I-J) The visceral endoderm thickening (VET) is used as a marker of the AVE region and is delineated by a white line. Examples of GFP-positive cells in wild type conceptuses, at E5.5L/D in I and E5.5D in J. Arrowheads point to the positive cells colonising the VET. I, J are green fluorescent projection of a confocal z series merged with a transmitted light section and I', J' are merged confocal sections. Scale bars 20 μm.

To investigate the origin of cells of the AVE, we performed a clonal analysis in the *Cer1-GFP *transgenic line, which marks cells of the AVE at E5.5. We injected surface cells of the ICM with an mRNA encoding a nuclear red-fluorescent protein. Only blastocysts with a single positive cell were transferred into the uterus of a foster mother. Conceptuses were collected at E5.5 and staged depending on the position of *Cer1-GFP *positive cells (Fig. 5D, G). The number of *Cer1-GFP *positive cells and the size of the conceptus at each stage was similar to non-injected conceptuses [19]. We found that 7 out of 29 clones of red-positive cells colonised *Cer1-GFP *expressing domain (Fig. 5B–C, E–F, H). In all cases these clones colonised only a subset of AVE cells, between 10 and 27%. To avoid the possibility that the lack of colonisation of the entire AVE is due to some cytotoxicity of the red marker (red clones were smaller, see Methods), we also carried out an alternative analysis by monitoring AVE cells based on their columnar shape [5]. Among the wild-type E5.5 conceptuses with labelled GFP cells described earlier, 34 were observed in an orientation which permits detection of the VET. In 17, GFP positive cells colonised the VET. In all these cases, only a fraction of the VET was labelled (Fig. 5I–J). Thus we conclude that the origin of the AVE is polyclonal. This is not only true in the blastocyst, but necessarily also for VE cells between E3.5 and E5.5. Indeed, if a single precursor of AVE existed at an intermediate stage, its mother cell in the blastocyst would necessarily contribute to the whole AVE in addition to other VE cells. Therefore the polyclonal origin of the AVE holds true from E3.5 to E5.5.

## Discussion

### Internalisation of the extra-embryonic ectoderm during egg cylinder formation

Systematic dissections from the uterus and analysis of early egg cylinder conceptuses have revealed a folding of the extra-embryonic ectoderm when the blastocyst transforms into the egg cylinder. This unexpected finding supports an earlier observation of "a slight depression on the surface of the trophoblast cone" which "becomes a funnel-shaped cavity", in sections of uterine horns containing conceptuses of similar appearance to those at the early egg cylinder stage in our study [20]. Based on the age of the conceptuses, we propose a model for the sequence of events leading to the formation of the extra-embryonic ectoderm. At pre-egg cylinder stage, polar trophectoderm cells are being rearranged. The localized accumulation of actin suggests that a contractile apparatus is assembled under the apical surface of polar trophectoderm cells. When it springs into action, it may contribute to the formation and the closure onto itself of the funnel-shaped depression. Later, at the early egg cylinder stage, no sign of actin enrichment or tissue depression remains, suggesting that the depression is occluded. It is only later, at E5.5L/D, that an extra-embryonic cavity forms, by extension of the embryonic pro-amniotic cavity [5,21]. This model is descriptive and determination of the precise mechanism of formation of the extra-embryonic region would require experiments on living embryos. Embryos at this early stage are still very difficult to culture and improvement of culture conditions will be necessary to investigate whether folding of the extra-embryonic ectoderm affects every embryo and to characterise further the nature of the contractile apparatus. From a phylogenetic point of view, the formation of a cup-shaped egg cylinder is a characteristic of mouse and some mammalian conceptuses, whereas others, including human or bovine, are planar [22]. This is thought to permit compaction of the volume of the conceptus in relation to the increase of litter size. Constriction of the polar trophectoderm might underlie this difference in conceptus shape.

Additional phenomena such as increased proliferation of cells from the polar trophectoderm [3], may contribute to the transformation of the blastocyst into the egg cylinder. Regional differences in cellular proliferation rates and mechanical constraints have been suggested to drive growth in the direction of least resistance, as if the extra-embryonic ectoderm was pushed towards the blastocoelic cavity [3]. This would also result in polar trophectoderm derivatives being engulfed by the VE. In this case, the VE of the extra-embryonic region may originate from cells initially covering the epiblast. These would come in contact with the extra-embryonic ectoderm as it is pushed into a territory previously occupied by the epiblast.

Conventionally, parietal endoderm is defined as the endoderm cells lining the inner surface of the mural trophectoderm [21]. Our model implies that some of these cells, after folding of the polar trophectoderm, might cover the extra-embryonic ectoderm and therefore adopt a position characteristic of the VE (Fig. 2G). This raises the question of where the limit between visceral and parietal endoderm is to be found. Different cell morphologies have been observed at day 5, such that parietal endoderm cells are stellate and form a discontinuous layer [23]. In our implanted blastocysts and pre-egg cylinder conceptuses (see Fig. 1E–F), we observe endoderm cells lining the mural trophectoderm, in continuity with the endoderm cells that contact the epiblast, and with a VE-like morphology. The identity of these cells is unclear. Even at a later stage, when both tissues have specific markers, cells at the junction between parietal and visceral endoderm, the so-called marginal zone, are ill-defined and show characteristics of both types [24]. Further studies will be required to understand how the primitive endoderm is segregated between parietal and visceral endoderm.

### The junction between embryonic and extra-embryonic VE regions is not a compartmental barrier during egg cylinder growth

The classical difference between VE cells in the extra-embryonic and embryonic regions is histological, the former having columnar shape and the latter a squamous shape [4]. We do not observe this difference at the early egg cylinder stage. It becomes only apparent from E5.5 with the exception of the columnar VET in the embryonic region [5]. Molecular differences are also observed, and were originally described at E6.5 based on the expression of genes such as those encoding the glycoprotein α-fetoprotein [6] or the activin receptor ActRIB [25]. It has recently been shown that specific expression of genes in the embryonic region of the VE is first visible at E5.25, whereas markers of the extra-embryonic region are down-regulated in the embryonic region at E5.5 [7].

We have found that clonally related VE cells may grow across the junction between embryonic and extra-embryonic regions from the early egg cylinder stage to E6.5. Previous studies have shown that, when AVE cells reach the embryonic/extra-embryonic junction, they transitorily spread along the junction [16] as if the junction could act as a transitory barrier to cell migration. AVE cells may occasionally cross the junction as they spread along it [5,16] and later, during gastrulation, are displaced into the extra-embryonic region by the forming definitive endoderm [26]. This indicates that while the junction affects AVE cell migration, it is not a compartmental barrier. Taken together these results show that embryonic and extra-embryonic VE regions are not distinct cellular compartments, despite their histological and molecular differences. This suggests that the differences between the embryonic and extra-embryonic regions are not related to the origin and nature of VE cells but are rather controlled by extrinsic signals from the underlying tissues, namely the epiblast and the extra-embryonic ectoderm. This is consistent with the finding that the expression of *α-fetoprotein *responds to an unknown signal from the extra-embryonic ectoderm [27].

### Distinct cell behaviour marks embryonic and extra-embryonic regionalisation of the visceral endoderm after AVE migration

Despite the lack of strict clonal restriction, cells of the VE display distinct growth modes in the embryonic and extra-embryonic regions by E6.5 [9,10]. We show that as soon as E5.5, coherent growth becomes specific to the extra-embryonic region whereas dispersion is enhanced in the embryonic region. The stage when these regionalised cell patterns appear is similar to that at which histological and molecular regionalisation becomes visible. Regionalised growth modes are still observed at later stages when VE in the embryonic region is being replaced by definitive endoderm during gastrulation [26]. It has been proposed that proteins of the extra-cellular matrix, hensin and laminin, mediate differences in the cell architecture of columnar and squamous VE [28]. Variations in the composition of the extra-cellular matrix may well underlie the distinct growth modes that we observe here by regulating cell behaviour such as intercalation, migration or division.

Interestingly, perturbation of the formation of the AP axis, by mutation in genes of the Wnt [29,30] or TGFβ [31] pathways inhibits the formation of distinct cell architecture. Nodal signalling, which plays a central role in AP axis specification [11], also affects the molecular regionalisation of the VE. Nodal is essential for the expression of genes, such as *Lhx1*, specific to the embryonic region of the VE and also to confine the expression of some markers, including *Furin*, to the extra-embryonic region [7]. These data suggest that regionalisation of the VE might be linked to the emergence of the AP axis as distinct behaviour of VE cells in the embryonic and extra-embryonic regions becomes pronounced only after migration of the AVE.

Migration of the AVE not only affects the regionalisation of VE cells, but also their behaviour at the embryonic/extra-embryonic junction. We have shown that orientation of labelled clusters at the level of the junction is shifted to become parallel to it. This may be due to new oriented cell division or to rearrangement of neighbouring cells. Cell labelling together with time-lapse observations at post-implantation stages have demonstrated that when AVE cells reach the anterior embryonic/extra-embryonic junction, they come to an abrupt halt and then start to spread laterally [5,16]. More distal embryonic VE cells are also displaced proximally and blocked at the level of the junction [5]. The transverse clusters observed on the embryonic side of the junction are most likely the result of this process. However, such clusters were also observed in the extra-embryonic region indicating that the distal to anterior movement of AVE cells restricts the exchange of cells between the extra-embryonic and embryonic regions in both directions. Importantly, this appears to be a specificity of the anterior pole, as vertical clusters can span the junction posteriorly.

### Polyclonal origin of the anterior visceral endoderm

We have assessed the origin of cells of the AVE using two criteria to identify this region: expression of a specific molecular marker and distinct morphology. We found that at E5.5 the *Cer1-GFP *expressing domain and the VET domain are never completely colonised by the descendants of one single surface ICM cell. These results show that the AVE has a polyclonal origin in the blastocyst.

The cellular composition of the AVE is unclear and several criteria are currently used to distinguish this region from the rest of the VE. These include the expression of specific molecular markers encoding transcription factors or secreted proteins from E5.5 to E6.5 (reviewed in [2]), its morphology as a localised columnar epithelium at E5.5 [5], the ability of cells to migrate from distal to anterior positions between E5.5 and E5.75 [15,16], a low proliferation rate [32] and its requirement for the maintenance of anterior epiblast and neurectoderm fates before and during gastrulation [12]. Cells in the AVE region seem to display heterogeneous characteristics already from E5.5 when they are first identified at the distal tip of the egg cylinder. Indeed at this stage, cells of the VET express *Hhex *in a salt and pepper pattern that does not completely overlap the expression domain of two other AVE markers, *Cer1 *and *Lefty1 *[16,32]. Mutations in the different genetic pathways involved in AVE specification affect differentially the expression of AVE markers, further highlighting the complexity of this region [11,33,34]. Whether the heterogeneity observed in the AVE region relates to possible distinct origins, fates and functions remains unknown.

The current view of how the AVE region is specified is that Nodal signalling from the epiblast is required for the formation of this specialised VE population at the distal tip of the E5.5 egg cylinder [11] and that a signal derived from the extra-embryonic ectoderm is necessary to prevent the ectopic expression of AVE markers in the rest of the VE [19,35]. Some genes marking the AVE at E5.5 like *Hhex, Cerl *and *Lefty1 *are already expressed in the E3.5 ICM and in the E4.5 primitive endoderm though with slightly different patterns [7,15,17,18,36]. Although the lineage of these cells is still unknown, these findings raise the question of whether AVE cells are specified during peri-implantation stages, before they can be morphologically identified at E5.5. Our results help to understand the origin of the AVE as they demonstrate that all AVE cells at E5.5 are not derived from a single surface ICM precursor in the E3.5 blastocyst. Whether there is a stage between E3.5 and E5.5 at which some VE cells will only give rise to descendants in the AVE remains to be understood. Further work will be required to clarify the timing and mechanisms of AVE specification. In particular it will be interesting to determine whether the complexity of the AVE cell population at E5.5 results from sequential steps of specification taking place during peri-implantation and early post-implantation development.

## Conclusion

We have analysed the formation of the embryonic and extra-embryonic regions of the conceptus as the blastocyst transforms into the egg cylinder. We show that the extra-embryonic region is clearly visible at the early egg cylinder stage and that its formation involves a folding of the extra-embryonic ectoderm. Using a cell labelling approach we find that the embryonic and extra-embryonic regions of the VE do not correspond to distinct cellular compartments, and that growth is not restricted across their junction even after the appearance of histological and molecular differences by E5.5. Our results suggest that the distinct characteristics observed in the embryonic and extra-embryonic regions of the VE are regulated by extrinsic signals, possibly from the adjacent epiblast and extra-embryonic ectoderm respectively, from E5.5. Thus, despite their common origin VE regions are characterised by distinct cell behaviour such that cell dispersion is more pronounced in the embryonic region after the migration of the AVE. At this time, VE cell clusters at the junction between the embryonic and extra-embryonic regions are reoriented to become parallel to it. Our clonal analysis of the AVE demonstrates for the first time that this anterior signalling centre arises from more than a single precursor between E3.5 and E5.5.

## Methods

### Mouse strains and conceptuses recovery

Conceptuses were collected from natural matings of F1 (C57BL/6xCBA) females with F1 males or transgenic *Histone2B-GFP *[37] or *Cer1-GFP *[38] males maintained on a regime of 12 hours dark and 12 hours light, the midpoint of the dark period being 1 a.m. All experiments with animals were conducted in accordance with UK Government Home Office Licensing regulations. Noon of the day of the vaginal plug was considered as day 0.5 of development (E0.5). Blastocysts were flushed from the uterus in M2 medium at about 8 a.m. (Fig. 1A) and cultured in KSOM in an atmosphere of 5% CO_2 _at 37°C. E4.7-E5.0 and E5.5 conceptuses were dissected from the decidua in DMEM supplemented with amino acids, HEPES 25 mM and 10% foetal calf serum. At E4.7-E5.0, the implantation site was visible as a slight bulge and the conceptus was collected in the crypt. E6.5 conceptuses were collected as previously described [9]. The parietal yolk sac, composed of mural trophectoderm and parietal endoderm cells [39], is highly autofluorescent and was removed for better observation of the underlying tissues.

### Preparation of synthetic capped mRNA

NlsGFP and nlsDsRed templates were cloned from pEGFP-N1 and pDsRedExpress (Clontech) into a modified pBluescriptRN3P vector [40]. Oligonucleotides NLS-F 5'-GATCCATGGCTCCAAAAAAGAAGAGAAAGGTA and NLS-R 5'-GATCTACCTTTCTCTTCTTTTTTGGAGCCATG containing a nuclear localisation signal between BamHI and BglII restriction sites, were annealed and ligated to a fragment of RN3P digested with BglII. This new NLS-RN3P vector was verified by sequencing. It was then cut with BglII and NotI and ligated to a BamHI-NotI fragment of pDsRedExpress or pEGFP.

For *in vitro *transcription, MmGFP [41], nlsGFP or nlsDsRed plasmids were linearised, and transcribed using the mMESSAGEmMACHINE kit (Ambion). Capped mRNA was purified from proteins with phenol/chloroform and from nucleotides on an Rneasy MinElute Cleanup column (Qiagen). For microinjection, mRNA was diluted at a final concentration of 0.1–0.3 μg/μl in RNAse-free water.

### Microinjection of single blastocyst cells

Microinjection was performed as previously described [9] on a LEICA DM-IRB inverted microscope equipped with micromanipulators and an Eppendorf transjector and connected to an electrometer. The needle was inserted through the mural trophectoderm and cells lying on the surface of the ICM were targeted. Blastocysts with positive ICM cells were screened about 1 hour after injection on an inverted microscope equipped with epifluorescence, and transferred into the uterus (1 to 8 blastocysts) of an E2.5 pseudopregnant mouse. A total of 2125 blastocysts were injected (Additional file 2A). The conceptuses analysed here contained labelled cells derived from precursors at random positions on the surface of the ICM. 891 were fluorescent positive and transferred; 566 post-implantation conceptuses were recovered out of which 220 were labelled.

Different labels were used for microinjection, MmGFP, nlsGFP and nlsDsRed. We have calculated the percentage of recovery of positive conceptuses for each of them (Additional file 2B). No difference was observed between the labels at E4.7-E5.0 (implanted blastocyst, pre-egg cylinder and early egg cylinder stages). However at E5.5, the percentage of recovered conceptuses for nlsDsRedExpress labelling was lower (27%) than for GFP (45%), indicating some cytotoxicity of nlsDsRedExpress compared to GFP, probably as a result of aggregation of nlsDsRedExpress over time. This was nonetheless the most efficient of 6 red markers that we have tested, in particular compared to its monomeric version mRFP1 [42].

To check the number of cells which had been labelled by microinjection, we scored a group of blastocysts (n = 36) under fluorescent microscopy 1–2h after the injection. We observed that 77% of positive blastocysts contained a single labelled cell (Additional file 2C). Blastocysts containing more than one positive cell most likely resulted from transmission of the label between clonally related cells as cytoplasmic bridges have been reported to exist after mitosis [43-45]. Alternatively, the injected cell may have divided shortly after injection. We demonstrated the existence of such bridges by injecting a group of blastocysts with mRNA encoding the membrane-targeted fluorescent protein gapRFP (Additional file 2D and 3, 4). Therefore, in most cases injection of a single ICM cell indeed corresponds to the labelling of a single cell or two sister cells.

Labelling of ICM cells is expected to give rise to epiblast, visceral endoderm and/or parietal endoderm descendants [46-48]. Due to technical limitations (see above), the contribution of labelled cells to the parietal endoderm has not been analysed in this study therefore we have focussed on their contribution to the VE. In some cases (23% at the early egg cylinder stage and 18% at E5.5), conceptuses with labelled VE cells also had labelled cells in the epiblast. We have pooled these cases, as distributions of VE cells were not different. At the early egg cylinder and E5.5 stages 20% of the conceptuses had labelled cells only in the epiblast. In some rare cases (7%) trophectoderm or extra-embryonic ectoderm cells were labelled. This is most likely due to accidental labelling of trophectodermal extensions lining the ICM surface. Specimens with labelled cells only in the epiblast or in the extra-embryonic ectoderm, with a low fluorescent signal in the VE or which were damaged during dissection, were not taken into account for subsequent analysis (Additional file 2A).

### Confocal analysis of post-implantation clones

Live conceptuses were observed and multi-channels (red/green/far red or transmission) multi-sections images were acquired on a BioRad Radiance 2100 confocal microscope with a 20X objective every 3–5 μm at E4.7-E5.0 and every 2 μm at E5.5. Scanning was performed on two opposite sides of the conceptus. Red E5.5 clones in *Cer1-GFP *conceptuses were scanned sequentially in red and green. In order to better visualise and count MmGFP positive cells, E5.5 conceptuses were also stained with the nuclear marker Draq5 (Biostatus limited) 10 μM for 15 min at 37°C. The size of conceptuses was measured using Photoshop or Image J millimetre.

### Phalloidin staining

Conceptuses obtained from natural matings of F1 (C57BL/6xCBA) females with transgenic *Histone2B-GFP *males [37] were fixed in 4% paraformaldehyde at 4°C overnight. They were rinsed in PBT, and stained for 1–2 hours in 0.5 μg/ml phalloidin-Texas red (Molecular Probes). Multi-channels (red/green) multi-sections images were acquired every 1 μm on a Perkin Elmer Spinning Disk Ultraview ERS confocal microscope with a 20X or 40Xoil objective.

### Statistical analysis

The statistical analyses were performed using the SPSS software. The non-parametric Mann-Whitney test and the Chi-square test were used. A probability of less than 0.05 was considered to indicate significance.

## Authors' contributions

APG and SMM designed and carried out the work, analysed the results and drafted the manuscript. APG produced most of the wild-type E5.5 labelled conceptuses. SMM performed the morphological study at E4.7-E5.0, produced the wild-type E4.7-E5.0 and *Cer1-GFP *E5.5 labelled conceptuses. KPN participated in the production of wild-type E5.5 labelled conceptuses. DG participated in testing various constructs and in the production of *Cer1-GFP *E5.5 labelled conceptuses. JC and MZG coordinated the project and critically revised the manuscript. MZG conceived, funded and supervised the project, which was carried out in her laboratory. All authors read and approved the final manuscript.

## Supplementary Material

Additional file 1 **Sequential stages in vivo for the formation of the extra-embryonic region of the egg cylinder**. (A, B) Example of a wild-type implanted blastocyst (A) which reached the pre-egg cylinder stage after 8h45 of in vitro culture (B). (C-F) Example of a H2B-GFP transgenic conceptus at the pre-egg cylinder stage (C-D) which developed to the early egg cylinder stage after 19h of in vitro culture (E-F). Bright field (A, B, C, E) and fluorescent images (D, F) are shown. Arrows point to the primitive or visceral endoderm, arrowheads to the polar trophectoderm. The contour of the epiblast (A-F) and of the extra-embryonic ectoderm (E-F) is highlighted in white. Insets in A-B show the shape of 3 neighbouring cells of the polar trophectoderm. In all images the mural trophectoderm is present but closely apposed to the primitive endoderm as the blastocoel collapsed. Conceptuses were cultured at 37°C in 5% CO2 and in DMEM medium containing non essential amino-acids, penicillin/streptomycin (Gibco) and 40% Human Cord Serum. Scale bar: 20 μm. Click here for file

Additional file 2 **Production of labelled conceptuses**. (A) Table summarising the injections carried out, by stage of recovery. EEC, early egg cylinder; D, distal; IB, implanted blastocyst; L, lateral; L/D, laterodistal; PEC, pre-egg cylinder. (B) Table comparing the rate of recovery of labelled conceptuses, according to the mRNA injected and the stage of recovery. (C, D) Examples of positive blastocysts after injection, showing a single positive cell (C) or two positive cells linked by a cytoplasmic bridge (D). (C) was injected with nlsGFP mRNA, whereas (D) is a control blastocyst injected with mRNA encoding the membrane-targeted fluorescent protein gapRFP (see also additional files 3, 4). Click here for file

Additional file 3 **Cytoplasmic bridges between ICM cells of the blastocyst**. Red-channel images of a blastocyst in which an ICM surface cell has been injected with gapRFP. Two positive sisters cells are visible, linked by a cytoplasmic bridge. A stack of 3 z-planes taken every 7.25 μm is shown. Click here for file

Additional file 4 **Cytoplasmic bridges between ICM cells of the blastocyst**. Merged (red and transmission channels) images of the same blastocyst as in the additional file 3. Click here for file
