# Supplementary figures and images for: Regionalisation of the mouse visceral endoderm as the blastocyst transforms into the egg cylinder
Source: BMC Dev Biol. 2007 Aug 16;7:96. doi: 10.1186/1471-213X-7-96 (PMC1978209; doi:10.1186/1471-213X-7-96)

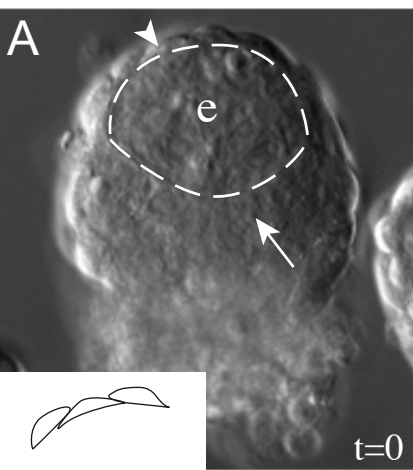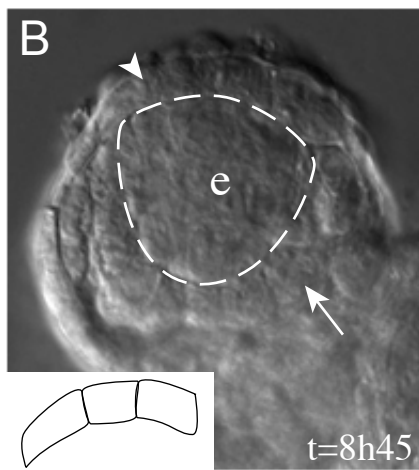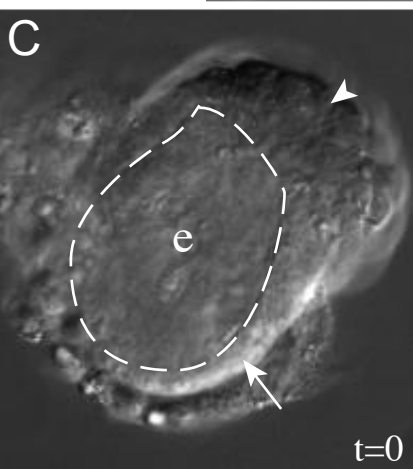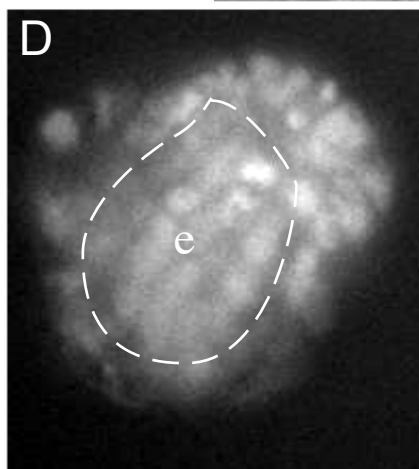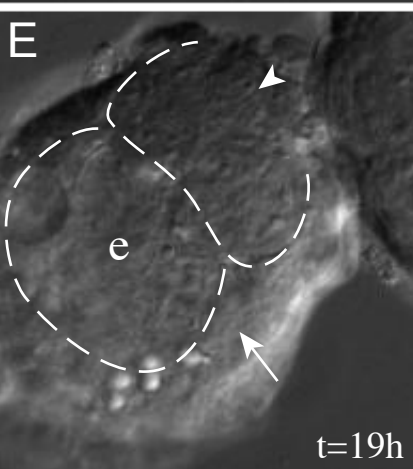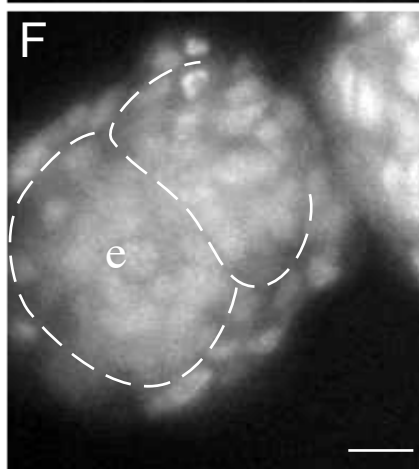

Supplement: Additional file 1 — Sequential stages in vivo for the formation of the extra-embryonic region of the egg cylinder. (A, B) Example of a wild-type implanted blastocyst (A) which reached the pre-egg cylinder stage after 8h45 of in vitro culture (B). (C-F) Example of a H2B-GFP transgenic conceptus at the pre-egg cylinder stage (C-D) which developed to the early egg cylinder stage after 19h of in vitro culture (E-F). Bright field (A, B, C, E) and fluorescent images (D, F) are shown. Arrows point to the primitive or visceral endoderm, arrowheads to the polar trophectoderm. The contour of the epiblast (A-F) and of the extra-embryonic ectoderm (E-F) is highlighted in white. Insets in A-B show the shape of 3 neighbouring cells of the polar trophectoderm. In all images the mural trophectoderm is present but closely apposed to the primitive endoderm as the blastocoel collapsed. Conceptuses were cultured at 37°C in 5% CO2 and in DMEM medium containing non essential amino-acids, penicillin/streptomycin (Gibco) and 40% Human Cord Serum. Scale bar: 20 μm. [file 1471-213X-7-96-S1.pdf]
