# Supplementary material for: Regionalisation of the mouse visceral endoderm as the blastocyst transforms into the egg cylinder
Source: BMC Dev Biol. 2007 Aug 16;7:96. doi: 10.1186/1471-213X-7-96 (PMC1978209; doi:10.1186/1471-213X-7-96)

A

Stage	Nb of blastocysts injected	Nb of positive blastocysts transferred	Nb of conceptuses recovered	Nb of labelled conceptuses recovered
E4.7-E5.0 (IB+PEC+EEC)	601	248	178	92 (24#)
E5.5 wt (D+L/D+L)	709	231	150	68 (48#)
E5.5 Cer	319	149	109	29
E6.5*	496	263	129	31
**Total**	**2125**	**891**	**566**	**220**

In brackets, nb of embryos analysed with labelled VE cells. Only E4.7-E5.0 conceptuses which had a clearly visible extra-embryonic region (EEC stage) were considered.

*Frequencies as in Weber et al., 1999

B

Injected mRNA	Stage	Percentage of labelled conceptuses among the recovered
nlsGFP	E4.7-E5.0	50%
nlsDsRed	E4.7-E5.0	53%
MmGFP	E5.5	45%
nlsDsRed	E5.5	27%

C

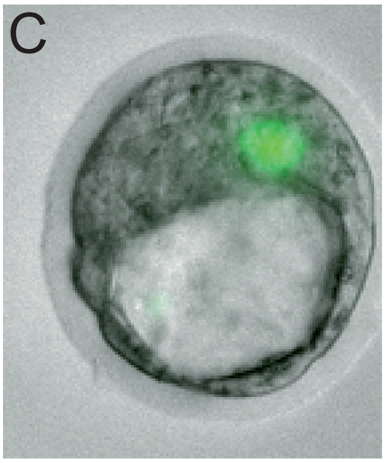

D

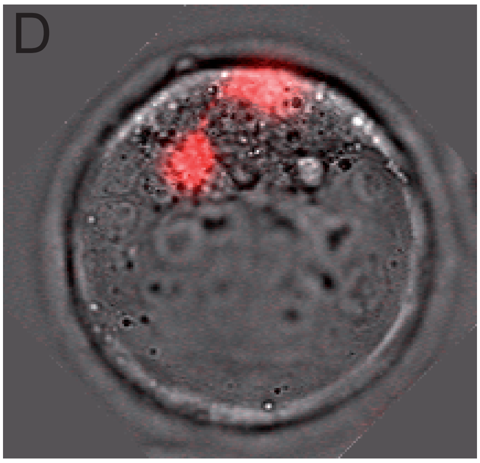

Supplement: Additional file 2 — Production of labelled conceptuses. (A) Table summarising the injections carried out, by stage of recovery. EEC, early egg cylinder; D, distal; IB, implanted blastocyst; L, lateral; L/D, laterodistal; PEC, pre-egg cylinder. (B) Table comparing the rate of recovery of labelled conceptuses, according to the mRNA injected and the stage of recovery. (C, D) Examples of positive blastocysts after injection, showing a single positive cell (C) or two positive cells linked by a cytoplasmic bridge (D). (C) was injected with nlsGFP mRNA, whereas (D) is a control blastocyst injected with mRNA encoding the membrane-targeted fluorescent protein gapRFP (see also additional files 3, 4). [file 1471-213X-7-96-S2.pdf]
